# Diffuse alveolar hemorrhage; An under-diagnosed and rare complication of glycoprotein IIb/IIIa inhibitors

**DOI:** 10.34172/jcvtr.2022.17

**Published:** 2022-06-25

**Authors:** Hamidreza Hekmat, Zahra Vahabi, Maryam Shojaeifard, Fatemeh Sadat Mirzadeh

**Affiliations:** ^1^Department of Cardiology, Ziaeian Hospital, School of Medicine, Tehran University of Medical Sciences, Tehran, Iran; ^2^Neurology Geriatric Department, Ziaeian Hospital, School of Medicine, Tehran University of Medical Sciences, Tehran, Iran; ^3^Echocardiography Research Center, Rajaie Cardiovascular Medical and Research Center, Iran University of Medical Sciences, Tehran, Iran; ^4^Department of Geriatric Medicine, Ziaeian Hospital, Tehran University of Medical Sciences, Tehran, Iran

**Keywords:** Glycoprotein IIb/IIIa Inhibitors, Alveolar Hemorrhage, Percutaneous Coronary Intervention, Acute Coronary Syndromes

## Abstract

Glycoprotein IIb/IIIa inhibitors play a key role in the treatment of patients who have acute coronary syndromes and undergone percutaneous coronary intervention. However, its serious complication is diffused alveolar hemorrhage. A 73-year-old diabetic woman presented with chest pain and dynamic ST elevation in ECG and positive troponin. She had occlusion in two coronary arteries and underwent percutaneous coronary intervention. The eptifibatide was administered. After hours, she showed respiratory symptoms, as well as drop of blood pressure and hemoglobin. All differential diagnoses suggested for her clinical presentation were evaluated, and finally, on the sixth day diffuse alveolar hemorrhage was diagnosed. Although respiratory symptoms such as hemoptysis and dyspnea may occur as complications of pulmonary edema and/or pneumonia, assumed clinical suspicion for pulmonary hemorrhage leading to early detection of it. Moreover, there is no definitive guideline for decreased bleeding complications and treatment of alveolar hemorrhage caused by glycoprotein IIb/IIIa receptor inhibitors.

## Introduction

 Eptifibatide blocks binding of fibrinogen and von-willebrand factor to glycoprotein IIb/IIIa receptor on platelet surface, and has Food and Drug Administration (FDA) approval for the treatment of Acute Coronary Syndromes (ACS). It is also recommended in patients who have undergone Percutaneous Coronary Intervention (PCI).^1^ In recent studies have shown the administration of GP IIb/IIIa inhibitors with both bivalirudin and UFH, occurring in selective conditions. The guidelines recommended GP IIb/IIIa inhibitors as the last option if evidence suggested no-reflow or a thrombotic condition. Although suggested the administration in patients who were not received P2Y12 receptor inhibitors before PCI because they were high-risk to hemorrhagic events. In these patients, the class/level of evidence GP IIb/IIIa inhibitors used at the time of PCI is IIb/C.^2^ Furthermore, this drug can diminish ischemic events after PCI if administered with aspirin and heparin.

 Glycoprotein IIb/IIIa inhibitors play a key role as potent antiplatelet agents; however, one of the important complications of these drugs is enhanced risk of bleeding, while their relation to Diffuse Alveolar Hemorrhage (DAH) is not well recognized.^3^ More recent studies indicate that DAH is rarely reported because it is underestimated. On the other hand, this complication has not been mentioned in the list of side effects of glycoprotein IIb/IIIa inhibitors.^3,4^

 Alveolar hemorrhage presentation could be misdiagnosed by other diseases such as acute pulmonary edema, a condition commonly seen in patients with ACS.^5^ Most cases of DAH are caused by capillaritis associated with systemic autoimmune diseases such as anti-neutrophil cytoplasmic antibody-associated vasculitis, anti-glomerular basement membrane disease, and systemic lupus erythematosus, but DAH may also result from coagulation disorders, drugs, inhaled toxins, or transplantation.^6^

## Case Presentation

**Figure 1:**
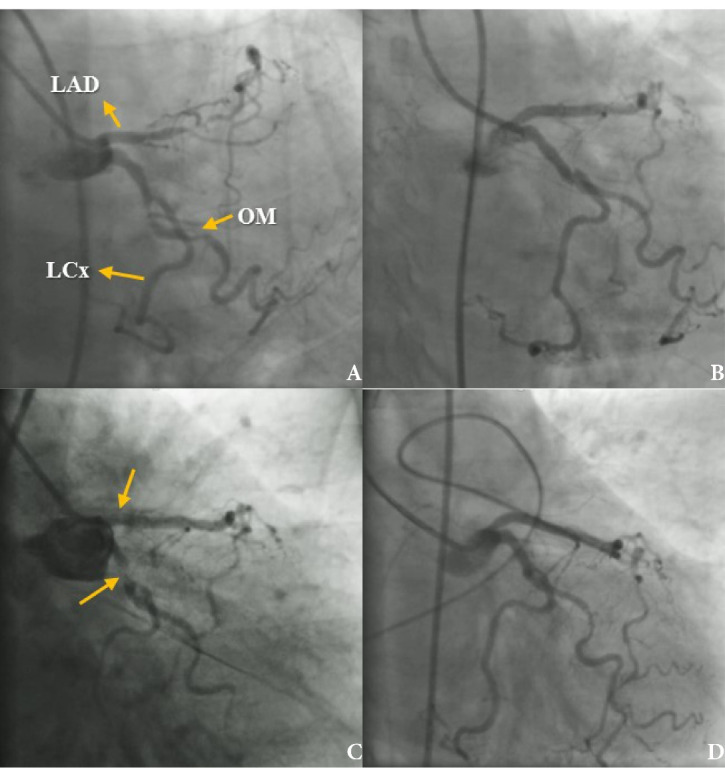
(A) Right anterior oblique (RAO) caudal view revealing a dominant left circumflex artery (LCX); several significant narrowing in LCX, obtuse marginal artery (OM), and left anterior descending (LAD); (B) After first percutaneous coronary intervention (PCI): implanted stent in left anterior descending (LAD) and left circumflex artery (LCx) to the obtuse marginal artery (OM), that showing these vessels were patent; (C) Coronary angiography revealing a large thrombus in the left circumflex artery (LCx) and left anterior descending (LAD); (D) reperfusion was performed successfully, again.

 A 73-year-old diabetic woman presented with chest pain and dynamic ST elevation in electrocardiogram and positive troponin. The patient was referred from another center for catheterization because she had recurrent angina and did had not consent to undergo Coronary Artery Bypass Surgery (CABG). Electrocardiogram in our hospital showed ST depression and T inversion in precordial leads. Angiography was performed which showed severe stenosis in Left Circumflex (LCx) and Left Anterior Descending artery (LAD). Also, there was no Right Coronary Artery (RCA) and LCx was dominant (Figure 1a). The drug-eluting stents were implanted into LCx and LAD. Complete reperfusion was achieved in the standard technique PCI (Figure 1b). Her vital sign was stable and had sinus rhythm in monitoring. Echocardiography was normal. She was transferred to the coronary care unit.

 Approximately 20 min after PCI, the patient developed chest pain and electrocardiogram monitoring showed ST elevation and 2:1 AV block. The patient was transferred immediately to the catheterization laboratory, where suddenly cardiac arrest occurred. Angiography and Cardiopulmonary Resuscitation (CPR) were performed simultaneously and pacemaker was implanted. Both stents of LCx and LAD formed thrombus, and LCx showed total occlusion (Figure 1c), and another time reperfusion was achieved through PCI (Figure 1d). Femoral access was used for both procedures. Eptifibatide was administered (180 mcg/kg IV stat, then continuous infusion 1 mcg/kg/min with another 180 mcg/kg IV bolus 10 minutes after 1st one). After CPR, the patient was admitted in the intensive care unit where hydration with N/S and dopamine infusion started.

**Figure 2:**
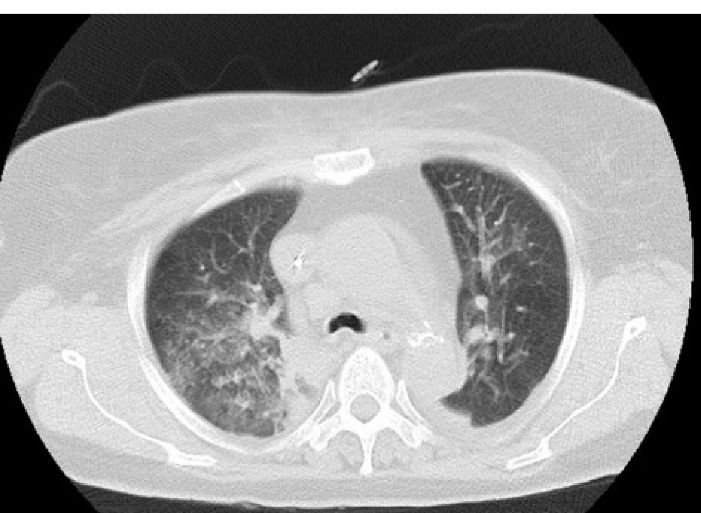
Areas of consolidation, ground-glass, and normal in pulmonary computed topography scan.

**Figure 3:**
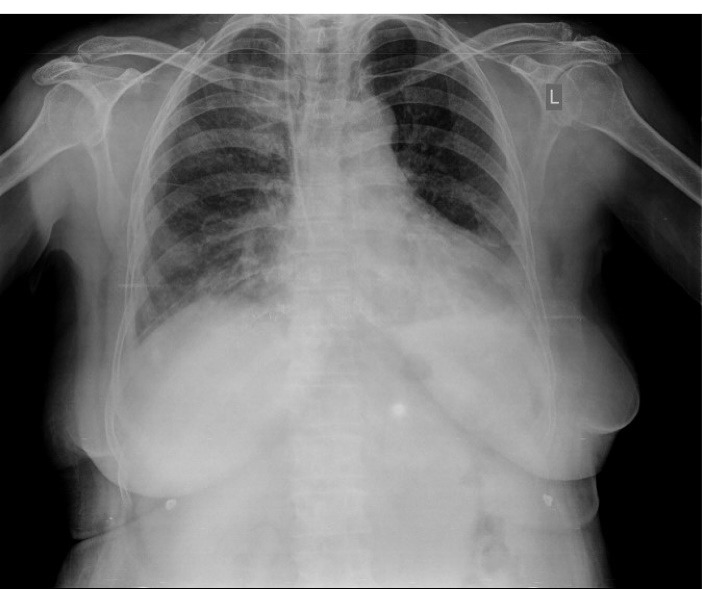
A chest X-ray eight days later shows improvement in alveolar infiltration.

 Three hours later, we noticed that she was pale and hypotensive. In physical examination, clear blood was seen inside the patient’s mouth but we did not find any bleeding signs in the nasal-gastric tube. Further, in the chest exam, she had diffuse rales over both lung areas, while there was no hemorrhagic site. Laboratory tests were performed and we found metabolic acidosis and drop of Hb from 13 to 7.5. With abdominal sonography, retroperitoneal hemorrhage was ruled out. Chest radiography exhibited bilateral alveolar infiltrates which was dominant in the right lung. A pulmonary computed tomography scan was done to rule out pneumonia, pulmonary edema, and pulmonary infarct (Figure 2). With suspected cardiopulmonary edema underwent LAD involvement to roll out of acute mitral regurgitation, repeated echocardiography was done that revealed mild LV dysfunction with mild MR. During the diagnostic evaluations, management steps were started simultaneously, including eptifibatide infusion discontinuation, administration of pack cell, and Fresh Frozen Plasma (FFP). After three days, the patient was extubated but developed a mild right hemiparesis, though brain MRI was normal and ischemic encephalopathy was raised. The patient found worsening hypoxia and alveolar infiltrates were diffused. Six days later based on these clinical manifestations and evaluation results, we suspected the diagnosis of DAH and performed diagnostic bronchoscopy. This revealed distributed dark blood throughout the bronchial tree and displayed on serial bronchiolar lavage hemorrhagic return was increased, thus confirming the diagnosis DAH. Figure 3 shows a chest X-ray eight days later, which alveolar infiltration improved. The patient had hemoptysis for 2 weeks. Also, she had functional loss in daily living activity. For rehabilitation and function improvement, she was transferred to the subacute geriatric ward. One month later, the patient was discharged with a relatively good general condition. Now, after 4 years, the patient is symptom free and on re-catheterization all the stents were patent.

## Discussion

 The estimated incidence rate of eptifibatide-induced DAH is 0.5%. The diagnosis of DAH relies on clinical suspicion combined with laboratory, radiologic, and pathologic findings. Early recognition is crucial, because prompt diagnosis and treatment is necessary for survival.^4^ Compared with pulmonary edema, pulmonary infarct, or pneumonia that occur as ACS or CPR complications, diagnosis of DAH is difficult and could be neglected due to its general symptoms, signs, and similarity of chest x-ray findings.^7^ In follow, incorrect treatment with loop diuretics or antibiotic therapy befalls by misdiagnosis.^8^

 The risk of bleeding increases with antithrombotic therapy and PCI. The evidence shows adverse outcomes such as mortality independently occurs with a higher risk in the patients with major bleeding. 40-60% of all bleeds in patients with ACS undergoing PCI are non-access-related bleeding events; that the risk of mortality in these events nearly two-fold higher than access-related bleeding events. 1.0% of patients undergoing PCI who treated with GP IIb/IIIa inhibitors have severe thrombocytopenia (defined as a platelet count of 20,000-50,000/mL).^2^ When the reversible binding GP IIb/IIIa inhibitors (eptifibatide) persist in circulation (half-life 2 h), the platelet transfusion can be inefficient in these patients, so Fresh frozen plasma (FFP) is used in patients with ongoing major bleeding.^9^

 It is generally considered safe to perform thrombolytic therapy if CPR is maintained for at least 10 minutes. However, there was no clear association between resuscitation duration and bleeding complications. ^10^ Meanwhile, when CPR was performed on this patient, no ribs were broken and as evident from the CXR images, there was no lung damage. Additionally, no clear correlation between CPR and DAH had been documented in the literature.^11^

 Diagnostic flexible bronchoscopy should be performed as soon as possible in patients with suspected DAH; more serious outcomes of DAH can be prevented by early diagnoses and following that appropriate treatment.^4,8^

 There are no unique guidelines for the treatment of DAH induced by GP IIb/IIIa receptor inhibitors.^3,4^ Since DAH can be unpredictable and lethal, the first line of treatment is to discontinue all anticoagulants and antiplatelets, correct coagulopathy, and protect the airway (mechanical ventilation).^4,8^ The benefits of using corticosteroids have not been established to treat drug-induced alveolar hemorrhage, including GP IIb/IIIa receptor inhibitors.^4^

## Conclusion

 In this case report, the patient showed unstable vital signs and hemoptysis after completing her cardiac procedure and once eptifibatide was administrated. Considering the vague symptoms and signs of DAH, a high degree of clinical suspicion is required for the diagnosis. Given these adverse outcomes such as considerable morbidity and mortality, further studies on diagnostic methods and treatment guidelines are recommended.

## Acknowledgements

 We highly appreciate our collaborators in CCU, ICU and geriatric wards for their kind readiness to help us in any circumstance.

## Funding

 There is no funding.

## Ethical approval

 The revealed report is nameless, and does not show the information of the patient. For the report of this data, informed consent was received from the patient. This Manuscript was writing according to the Committee on Publication Ethics (COPE).

## Competing interest

 The authors declare that they have no conflict of interest.
